# Percolation-Triggered Negative Permittivity in Nano Carbon Powder/Polyvinylidene Fluoride Composites

**DOI:** 10.3390/molecules29163862

**Published:** 2024-08-15

**Authors:** Guangyue Shi, Xiaolei Sun, Yao Liu

**Affiliations:** 1 School of Materials Science and Engineering, Tianjin Key Laboratory for Rare Earth Materials and Applications, Center for Rare Earth and Inorganic Functional Materials, Nankai University, Tianjin 300350, China; 2 Key Laboratory for Liquid-Solid Structural Evolution and Processing of Materials (Ministry of Education), Shandong University, Jinan 250061, China

**Keywords:** polyvinylidene fluoride, nano carbon powder, percolating composites, electrical properties, negative permittivity

## Abstract

Percolating composites exhibiting negative permittivity have garnered considerable attention due to their promising applications in the realm of electromagnetic shielding, innovative capacitance devices, coil-less inductors, etc. Nano carbon powder/polyvinylidene fluoride (CP/PVDF) percolating composites were fabricated that exhibit Drude-type negative-permittivity behavior upon reaching the CP percolation threshold. This phenomenon is attributed to the formation of a plasmonic state within the interconnected CP network, enabling the delocalization of electrons under the alternating electric field. Furthermore, a significant (nearly two orders of magnitude) increase in the conductivity of sample is observed at a CP content of 12.5 wt%. This abrupt change coincides with the percolation phenomenon, suggesting a transition in the conduction mechanism. To elucidate this behavior, comprehensive analyses of the phase composition, microstructure, AC conductivity, and relative permittivity were performed. Additionally, the sample containing 5 wt% CP exhibits a remarkably high permittivity of 31.5, accompanied by a relatively low dielectric loss (tanδ < 0.2). The findings expand the potential applications of PVDF, while the fabricated percolating composites hold promise for electromagnetic shielding, antennas, and other electromagnetic devices.

## 1. Introduction

The negative permittivity ($\varepsilon_{r}^{\prime }$ < 0), commonly defined as the condition where the real part of the relative permittivity is negative, serves as a cornerstone of metamaterial research, holding immense promise for applications in meta-antennas, unconventional lenses, and wave absorption [1,2,3]. This exotic property arises from the incorporation of meticulously designed periodic structures within metamaterials. The resulting effective permittivity can be actively tuned by manipulating the size, shape, or geometrical arrangement of these constituent units. However, the fabrication of such intricate periodic architectures often presents significant challenges, including complexity and high cost. To address these limitations, researchers have explored alternative approaches utilizing random percolating composites. This strategy successfully achieves negative permittivity through the controlled distribution of conductive and dielectric components within the composite. Importantly, the resulting negative permittivity remains tunable [4,5]. By strategically modifying the functional phase content or chemical composition of these composites, researchers can achieve a high degree of design flexibility, enabling the creation of novel metamaterial functionalities. This newfound tunability paves the way for the development of next-generation metamaterial devices with an optimized performance [6,7,8].

Percolating composites, a class of composite materials, are characterized by a dispersed conductive phase embedded within a continuous insulating matrix phase. Conductive fillers, such as metals, carbon materials, and intrinsically conducting polymers, can be incorporated to achieve specific electrical properties [9,10]. Wei et al. explored this concept by fabricating copper/titanium dioxide composites. As the copper content exceeded the percolation threshold, the resulting composites demonstrated negative permittivity at low frequencies. This phenomenon can be ascribed to the delocalization of electrons within the percolating copper network [11]. Similarly, Song et al. synthesized percolating composites using ZrO_2_/C nanoparticles, derived from metal–organic frameworks (MOFs), as the conductive phase and polyvinylidene fluoride (PVDF) as the matrix. With ZrO_2_/C content approaching 30 wt%, a distinct transition from hopping to metallic conduction was observed. This transition, accompanied by negative permittivity, signifies the establishment of a conductive network throughout the composite, confirming the occurrence of electrical percolation [12]. Fan’s group investigated the weak negative permittivity of graphene–carbon black/CaCu_3_Ti_4_O_12_ percolating composites. With 8%~10% graphene–carbon black, the composites exhibited weak negative dielectric properties stemming from the electrical dipole resonance in the functional phase, corresponding to the Lorentz model. Upon further increasing the functional phase content to 14%~16%, the negative permittivity intensified with the increasing functional-phase content, originating from plasma resonance within the three-dimensional conductive network, corresponding to the Drude model [13]. Consequently, the negative-permittivity behavior of percolating composites strongly depends on the conductive functional fillers. This dependence can be attributed to the excitation of delocalized electrons within the conductive fillers, leading to a phenomenon known as plasma oscillation. This phenomenon is a key contributor to the emergence of negative permittivity in these materials. However, the use of metals with high intrinsic electron density as the conductive phase often results in large negative-permittivity values (up to the order of 10^5^), thus hindering the precise control and practical application of negative-permittivity properties. Additionally, metals have high percolation thresholds and high intrinsic densities, posing challenges for the fabrication of percolation composites. Therefore, researchers are increasingly turning their attention to carbon materials, which possess advantages such as a light weight, chemical stability, and, more importantly, diverse morphologies, enabling lower percolation thresholds and negative-permittivity values, and ultimately allowing for finer control of negative-permittivity performance [14,15,16].

Our investigation leverages nano carbon powder (CP) as the conductive phase due to its high specific surface area. This zero-dimensional nanomaterial offers a unique opportunity to explore the influence of carbon morphology on the electromagnetic properties of percolating composites and their negative-permittivity response. Notably, the selection of nano carbon powder aligns with the growing interest in understanding the role of various carbon nanostructures in regulating percolation behavior. Polyvinylidene fluoride (PVDF) is chosen as the dielectric matrix owing to its exceptional chemical stability. Furthermore, the inherent piezoelectric properties and high dielectric constant of PVDF make it a well-established choice for electromagnetic composites [17]. This combination of materials offers a promising platform for investigating the interplay between nano carbon morphology, percolation network formation, and the resulting negative permittivity in these composites.

In this work, nano carbon powder/polyvinylidene fluoride (CP/PVDF) percolating composites were fabricated via a hot-pressing technique. The CP/PVDF composites exhibited negative permittivity once the CP content surpassed the percolation threshold. Notably, composites with CP content below the percolation threshold demonstrated a relatively high permittivity and low dielectric loss. The underlying mechanisms governing the dielectric and conductive behavior were subsequently elucidated in detail.

## 2. Results and Discussion

### 2.1. Phase Composition and Microstructure

The fabrication of CP/PVDF composites is described in the Experimental Section. In essence, a high-energy ball-milling process was employed to thoroughly mix nano carbon powder and PVDF powder, followed by hot pressing to consolidate the mixture into a form suitable for electrical measurement, using an LCR meter. Based on the mass fraction of nano carbon powder in the samples, we labeled the samples as follows: 0, 2.5, 5, 7.5, 10, 12.5, 15, and 17.5 wt%.

**Figure 1:**
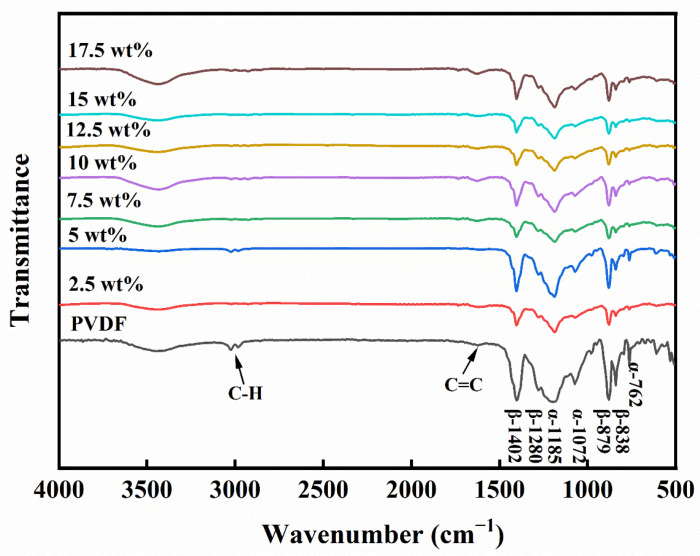
FTIR spectra of the PVDF and CP/PVDF composites.

The FTIR spectrum in Figure 1 delineates the compositional fingerprint of PVDF and CP/PVDF composites, with distinct peaks attributed to the α and β phases of PVDF. The peak observed at 3400~3500 cm^−1^ originates from the stretching of O-H, which could be attributed to moisture in the PVDF powder or water absorbed by the sample during the preparation process for the FTIR test. The peak near 1627 cm^−1^ originates from the C=C bonds present in both PVDF and nano carbon powder. Additionally, the spectral region from 2850 to 3000 cm^−1^ is characterized by peaks arising from the vibrational modes of the C-H bonds within the PVDF matrix [18]. For the α phase, the 762 cm^−1^ absorption peak is indicative of the molecule’s in-plane motion and wagging, the 1072 cm^−1^ peak signals the rocking vibration of the CF_2_ group, and the 1185 cm^−1^ peak corresponds to the C-F bond’s stretching. In the β phase, the 1402 cm^−1^ peak is associated with the CH_2_ group’s rocking vibration, the 1280 cm^−1^ peak is linked to the all-trans TT structure’s vibration within PVDF, the 879 cm^−1^ peak is attributed to the symmetrical stretching vibration of the CF_2_, and the 838 cm^−1^ peak denotes the asymmetrical stretching vibration of CF_2_. Notably, the dual peaks at 879 cm^−1^ and 838 cm^−1^ are characteristic of the all-trans TT structure in PVDF, underscoring the spectrum’s utility in elucidating the molecular dynamics and phase characteristics of the composites [19,20,21]. According to the FTIR test results, the main crystal structures of the PVDF powder used were the α phase and the β phase.

**Figure 2:**
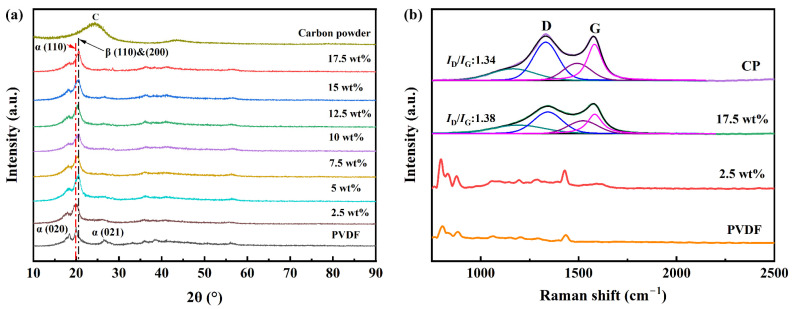
(**a**) XRD spectra. (**b**) Raman spectra of CP, PVDF, and CP/PVDF composites, with the spectra of CP and 17.5 wt% samples deconvoluted into four peaks: D (blue), TPA (green), A (purple), and G (pink).

The phase compositions of PVDF, nano carbon powder, and CP/PVDF composites were investigated using X-ray diffraction (XRD) and Raman spectroscopy. The XRD patterns in Figure 2a confirm the coexistence of α and β phases in the PVDF powder. Three prominent diffraction peaks are evident at 2θ = 18.8°, 2θ = 20.2°, and 2θ = 26.6°. These peaks correspond to the (020), (110), and (021) crystal planes of the α-phase, respectively. The diffraction peak at 2θ = 20.6° represents a superposition of the (110) and (200) crystal planes of the β-phase [22]. The XRD pattern of nano carbon powder exhibits a broad hump in the range of 20°~25°, which is characteristic of amorphous carbon [23,24]. This hump overlaps with the diffraction peak of PVDF molecules at 2θ = 26.6°. It is noticeable that, with the increasing CP content, there is a slight shift in the peaks of the XRD. This shift may be attributed to the addition of nano carbon powder, which interacts with the polymer matrix, affecting the crystal structure of the polymer and consequently causing a slight shift in the peak positions [25,26].

Raman spectroscopy, as shown in Figure 2b, further elucidates the phase composition of the composites. When the nano carbon-powder content is low, the Raman spectrum of the sample resembles that of PVDF. The peak at 1433 cm^−1^ is still observable in the 2.5 wt% CP sample, corresponding to the C-H bond stretching vibration of PVDF molecules [27]. With increasing nano carbon-powder content, the Raman spectrum simplifies, exhibiting only two characteristic peaks: a D band at 1340 cm^−1^; and a G band at 1570 cm^−1^, commonly associated with carbon materials. The D band originates from amorphous carbon and structural defects within nano carbon powder, while the G band arises from the C-C stretching vibrations of sp^2^ hybridized carbon atoms in graphitic carbon [28]. For a more precise analysis of the D and G peaks, the Raman spectra of nano carbon powder and the 17.5 wt% sample are deconvoluted into four peaks, as shown in Figure 2b. For both the carbon powder and the sample, the *I*_D_/*I*_G_ values are 1.34 and 1.38, respectively, revealing the presence of numerous defects [29]. These Raman spectroscopy results indicate that, as the nano carbon-powder content increases, the characteristic peaks of PVDF gradually become obscured, and only the characteristic peaks of nano-carbon black become apparent in the Raman spectrum of the sample.

**Figure 3:**
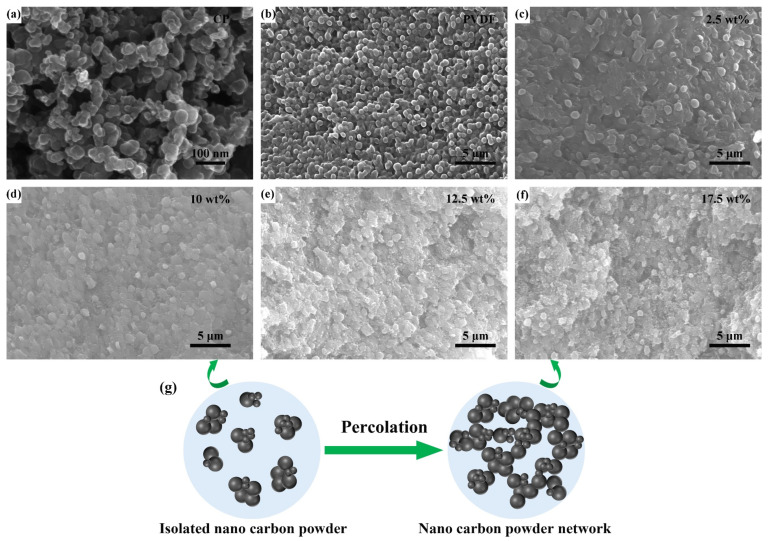
SEM images of the CP/PVDF composites, (**a**) nano carbon powder, (**b**) PVDF matrix, (**c**) 2.5 wt%, (**d**) 10 wt%, (**e**) 12.5 wt%, and (**f**) 17.5 wt%. (**g**) Schematic diagram of percolation behavior of the CP/PVDF composites.

Figure 3a–f present SEM images of nano carbon powder, PVDF matrix, and CP/PVDF composites with varying carbon-powder contents (2.5, 10, 12.5, and 17.5 wt%), respectively. As illustrated in Figure 3a, the nano carbon powder used in the experiment has a particle size ranging from 30 to 50 nm. It is also worth noting that the carbon powder exhibits significant agglomeration in its natural state, which necessitates the use of high-energy ball milling to uniformly mix the carbon powder with PVDF powder during the sample-preparation process. Figure 3b displays the cross-sectional morphology of the matrix, where the PVDF has formed a relatively dense structure following the hot-pressing method. At the 2.5 wt% content level (Figure 3c), the CP appears as isolated particles dispersed within the PVDF matrix. As the content increased to 10 wt% (Figure 3d), some agglomerates of carbon powder begin to form. However, these clusters remain largely isolated. The noticeable morphological changes in the nano carbon powder can be observed in Figure 3e. Here, the CP clusters begin to interconnect, forming a rudimentary three-dimensional network throughout the composites. This network becomes more extensive with a further increase in CP content to 17.5 wt% (Figure 3f). The observed microstructural evolution strongly suggests the occurrence of a percolation phenomenon for CP within the CP/PVDF composites at a critical loading between 10 and 12.5 wt%. Figure 3g offers a clearer depiction of the percolation dynamics in the CP/PVDF composite materials. When the functional-phase content is below the percolation threshold, the nanoparticles and their formed aggregates are isolated and dispersed within the matrix. As the functional-phase content increases and exceeds the percolation threshold, the nanoparticles become widely distributed and interconnected within the matrix, forming a three-dimensional network. The structural significance of conductive carbon path disconnection and connection, collectively termed percolation behavior, extends far beyond mere structural considerations. Percolation signifies the formation of interconnected pathways through the CP phase, enabling potential electrical conductivity throughout the composites.

### 2.2. AC Conductivity

**Figure 4:**
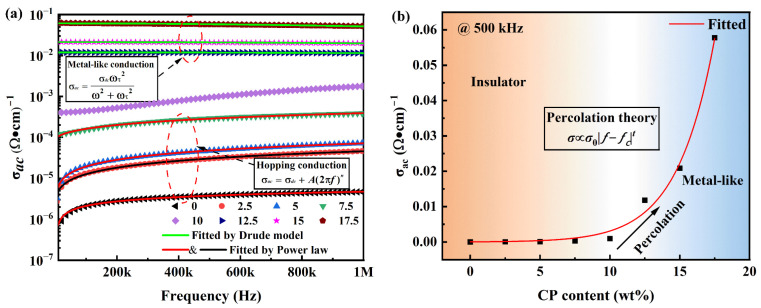
(**a**) Frequency dependences of ac conductivity of CP/PVDF composites. (**b**) Variation in AC conductivity with CP content at 500 kHz.

Figure 4a reveals a positive correlation between AC conductivity and frequency for CP/PVDF composites containing less than 10 wt% nano carbon powder. However, the conductivity values remain relatively low within this range. This behavior aligns with the power-law relationship typically observed in samples with low functional-phase content (i.e., below 10 wt%). The power-law model accurately describes the frequency dependence of electrical conductivity in samples containing low amounts of nano carbon powder [30]. The expression for this relationship is given as follows:

(1) $$\sigma_{\mathrm{ac}}=\sigma_{\mathrm{dc}}+A{\left(2\pi f\right)}^{n}$$

where σ_dc_ represents the DC conductivity, *A* is a constant coefficient, *f* denotes the frequency, and *n* signifies the exponent factor. The power-law model frequently serves as a valuable tool for elucidating the frequency dependence of AC conductivity in composite systems characterized by high degrees of disorder [31]. As depicted in Figure 4a, the excellent agreement between the power-law fitting curves (red solid lines) and the experimental data underscores the applicability of this model for composites with low functional-phase content (below 10 wt%). This alignment suggests a dominance of the hopping conduction mechanism in these sparsely filled composites, further implying their insulating behavior.

As the content exceeds 12.5 wt%, the AC conductivity significantly increases, while the conductivity decreases with the increasing frequency, obeying the free electron theory, also known as the Drude model [32].

(2) $$\sigma_{\mathrm{ac}}=\frac{\sigma_{\mathrm{dc}}\omega_{\tau }^{2}}{\omega^{2}+\omega_{\tau }^{2}}$$

where *ω*_τ_ is the collision frequency. As depicted in Figure 4a, the green solid lines illustrate the fitting results based on the Drude model. This agreement suggests a transition toward metal-like conductivity in CP/PVDF composites with carbon powder loadings exceeding 12.5 wt%. This behavior aligns with the concept of conductor behavior, signifying a substantial increase in electrical conductivity. The observed shift in the dominant conduction mechanism can be attributed to the well-established phenomenon of the percolation. Above this critical concentration, the formation of interconnected conductive networks within the composite facilitates efficient charge transport, leading to a conductor-like response.

The variation in AC conductivity with CP content at 500 kHz is shown in Figure 4b. In samples with carbon-powder contents of 12.5 wt% and higher, the AC conductivity increases significantly, with values from 2 to 3 orders of magnitude greater than those of samples with 10 wt% or less. In conjunction with the previous analysis, the percolation threshold is between 10 and 12.5 wt%. The observed increase in electrical conductivity suggests the establishment of a percolating network within the composites and can be described by percolation theory [33].

(3) $$\sigma \propto {\left|{f-f}_{c}\right|}^{t}$$

where *f* represents the CP fraction, and *f_c_* denotes the percolation threshold for the CP/PVDF composites. The red solid line in Figure 4b represents the fitting results obtained using percolation theory. The fitted data coincide with the experimental results, further corroborating the occurrence of the threshold-penetration phenomenon.

In brief, for the CP/PVDF composites, when the content exceeds the threshold, percolation occurs, and the nano carbon powder particles contact each other, forming a three-dimensional conductive network and expanding to the whole sample, thus leading to a change in the conductive mechanism and an increase in the AC conductivity.

### 2.3. Reactance Analysis

**Figure 5:**
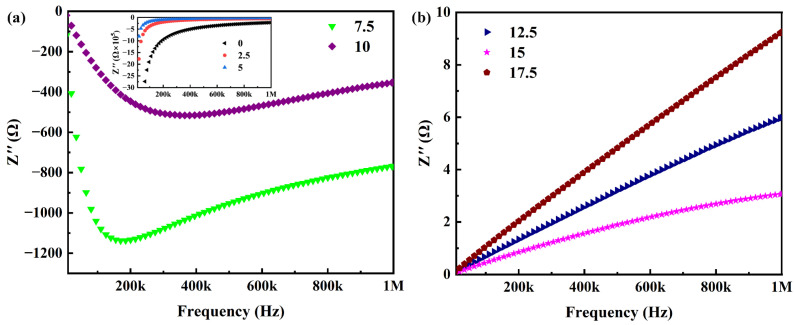
Reactance spectra of the CP/PVDF composites. (**a**) Samples with CP content below percolation threshold, (**b**) samples with CP content exceeding percolation threshold.

The impedance–frequency spectroscopy data presented in Figure 5a unveil a critical aspect of the CP/PVDF composites with functional phase content below the percolation threshold. These samples exhibit negative reactance values at lower frequencies. This observation signifies that the internal current phase leads to the applied voltage phase, a characteristic response of capacitive materials. Interestingly, the reactance behavior of the 7.5 wt% and 10 wt% CP samples deviates from this trend. Their reactance spectra initially exhibit an increase followed by a decrease with increasing frequency. This unique response suggests the coexistence of both capacitive and inductive components within the material. The emergence of these inductive contributions can be attributed to the formation of localized conductive networks arising from the agglomeration of carbon powder particles.

Conversely, Figure 5b shows that the impedance–frequency response of the composites surpasses the percolation threshold. In this case, the measured reactance values are positive across the entire frequency range. This positive reactance signifies that the internal voltage phase leads to the current phase, a behavior characteristic of inductive materials. It is noteworthy that the sample containing 15 wt% exhibits the lowest impedance value, which may stem from the composites’ dielectric properties being sensitively dependent on the microstructure. Samples with varying contents of functional phases possess distinct internal microstructures; in the case of the 15 wt% sample, the lower impedance is likely due to the formation of a more interconnected percolation network within [34].

In essence, the combined observations from Figure 5a,b illustrate a percolating phenomenon. As the CP content within the CP/PVDF composites transitions beyond the percolation threshold, a distinct shift in the electrical response is observed. The composites exhibit a transition from capacitive to inductive character, a hallmark response associated with materials exhibiting negative permittivity [35].

### 2.4. Negative-Permittivity Analysis

**Figure 6:**
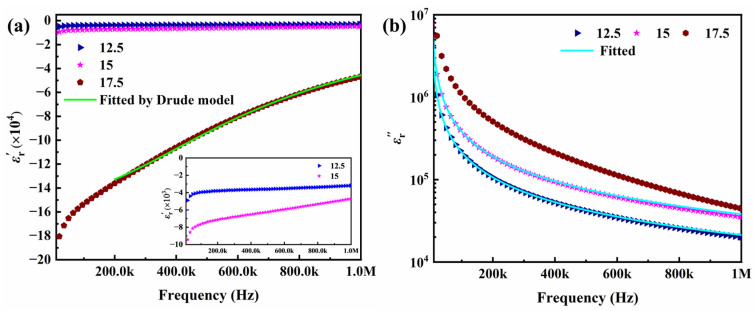
Frequency dependence of the complex permittivity for the CP/PVDF composites exceeding the *f*c: (**a**) real part and (**b**) imaginary part.

As illustrated in Figure 6, the real part of relative permittivity exhibits a negative value when the nano carbon powder loading surpasses the percolation threshold. The absolute value of the negative permittivity increases with increasing CP content. In conjunction with the analysis of the reactance behavior of the material in Section 2.3, the negative-permittivity behavior is associated with the inductive characteristics exhibited by the composites. A further analysis of the negative-permittivity behavior exhibited by the composites revealed that the Drude model in free electron theory can be used to fit the trend of the negative permittivity with frequency. The Drude-model formula is as follows [36]:

(4) $$\varepsilon_{r}^{\prime }\left(\omega \right)=1-\frac{\omega_{p}^{2}}{\omega^{2}+\omega_{\tau }^{2}}$$

(5) $$\omega_{p}=\sqrt{\frac{n_{eff}e^{2}}{m_{eff}\varepsilon_{0}}}$$

where *ω* represents the angular frequency (*ω* = 2π*f*), *ω*_p_ denotes the plasma frequency, and *ω*_τ_ signifies the collisional frequency, which is also the inverse of the relaxation time (*ω*_τ_ = 1/τ). Additionally, *n_eff_* refers to the effective concentration of free electrons, *m_eff_* represents the effective mass of the electron, and *ε*_0_ denotes the vacuum permittivity. Figure 6a presents the fitting results for the negative permittivity of the 17.5 wt% carbon-powder sample using the Drude model (green solid line). This fitting process yields plasma frequency ($\omega_{p}$) and collision frequency ($\omega_{\tau }$) values of 2.58 × 10⁸ rad/s and 6.8 × 10⁵ rad/s, respectively. With a reliability factor as high as 0.998, free electron theory emerges as a powerful tool for understanding the negative permittivity in CP/PVDF composites. The observed negative permittivity can be understood within the framework of percolation theory and the formation of conductive networks. When the carbon-powder content surpasses the percolation threshold, the interconnectivity between nano carbon powder establishes a continuous three-dimensional conductive network. This network houses a significant population of free electrons. Under the influence of an external alternating electric field, these free electrons experience a restoring electrostatic force but also possess inertia, leading to reciprocating motion along the direction of the field. At a specific frequency, a resonance phenomenon known as plasma oscillation occurs within the free electron population. This collective oscillation manifests as a negative permittivity. Furthermore, Equations (4) and (5) elucidate a positive correlation between the absolute value of the real part of the relative dielectric constant and the concentration of free electrons. As demonstrated in Figure 6a, the absolute value of the real part increases with the rising content of nano carbon powder. This increase in the CP content is what leads to the augmentation in the concentration of free electrons. This relationship suggests that the negative permittivity of percolating composites can be precisely controlled by tailoring the content of the conductive phase in the composites.

The imaginary part ($\varepsilon_{r}^{\prime \prime }$) of the relative permittivity is a key parameter for quantifying dielectric loss within materials. As illustrated in Figure 6b, for composites exceeding the percolation threshold for functional phase content, $\varepsilon_{r}^{\prime \prime }$ exhibits a dependence on frequency. This loss can be attributed to two primary mechanisms: polarization loss ($\varepsilon_{p}^{\prime \prime }$), arising from the interaction of the electric field with polarizable components; and conductive loss ($\varepsilon_{C}^{\prime \prime }$) associated with the movement of charges within the composite. The following equation expresses the relationship between these contributions and the total imaginary permittivity [37]:

(6) $$\varepsilon_{r}^{\prime \prime }=\varepsilon_{C}^{\prime \prime }+\varepsilon_{P}^{\prime \prime }$$

Furthermore, $\varepsilon_{C}^{\prime \prime }$ can be expressed as follows:

(7) $$\varepsilon_{C}^{\prime \prime }=\frac{\sigma_{\mathrm{dc}}}{2\pi f\varepsilon_{0}}$$

where σ_dc_ denotes the DC conductivity. From Equation (7), the conductive loss of the composites is negatively correlated with the frequency, i.e., $\varepsilon_{C}^{\prime \prime }\propto 1/f$. As shown in Figure 6b, the imaginary component ($\varepsilon_{r}^{\prime \prime }$) of the permittivity for all three samples exhibits a decreasing trend with increasing frequency. This behavior is consistent with the dominance of conductive loss mechanisms within the composites. To quantify this behavior, Equation (7) was used to fit the $\varepsilon_{r}^{\prime \prime }$ spectra of the samples containing 12.5 and 15 wt% carbon powder. The excellent agreement between the fitted curves (green solid lines) and the experimental data underscores the validity of this approach. This alignment suggests that internal conductive loss is the primary contributor to the overall dielectric loss in these composites with high carbon-powder loadings. As can be further observed from the figure, the imaginary part of the relative permittivity increases with increasing nano carbon-powder content, indicating that the loss of the composites increases with increasing functional phase content. This is because the increased content of the conductive functional phase leads to the expansion of conductive pathways, resulting in increased conductive loss.

Table 1 presents a comparison of the negative-permittivity properties of CP/PVDF composites with other polymer-based percolating composites using nano carbon materials as functional phases. The filler contents listed represent the amounts of functional phases just exceeding the percolation threshold, while the negative-permittivity values correspond to the dielectric constants measured at these concentrations. Research is mainly focused on the frequency range from kHz to MHz. Owing to the diverse morphologies of nano carbon materials, percolating composites utilizing different carbon materials exhibit considerable variations in their percolation threshold and negative permittivity. Notably, the percolation threshold in this study is higher compared to composites employing graphene and carbon nanotubes as functional phases. This phenomenon can be attributed to the morphology of the zero-dimensional nano carbon powder. Due to its particle-like morphology and small three-dimensional scale, a higher content of CP is required to form a conductive network in the PVDF matrix, resulting in a higher percolation threshold for the CP/PVDF composites. This indicates that precise control over negative-permittivity performance can be achieved by selecting different types of carbon materials or by managing the content of the functional phase.

### 2.5. High Permittivity Analysis

**Figure 7:**
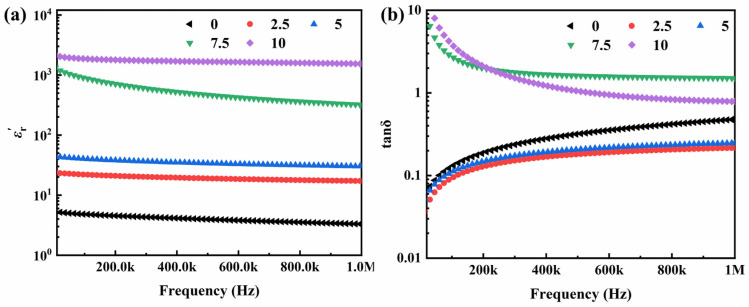
(**a**) Frequency dependence of the real part of the complex permittivity for CP/PVDF composites with CP content below percolation threshold. (**b**) The dielectric loss tangent (tanδ) of the CP/PVDF composites.

PVDF exhibits a high dielectric constant ranging from 5 to 9, surpassing that of other polymer materials, making it a widely employed material for high-permittivity research [39]. Beyond the negative-permittivity properties, we also investigated the high-permittivity behavior of CP/PVDF composites with nano carbon-powder content below the percolation threshold. The dielectric properties of the CP/PVDF composites with carbon-powder contents of 10 wt% and lower are shown in Figure 7a. The dielectric constant of the PVDF material employed in this experiment is approximately 5. Upon the incorporation of nano carbon powder, the dielectric constant of the CP/PVDF composites is enhanced, demonstrating a continuous increase with increasing functional-phase content. This enhancement in the dielectric constant is attributed to the addition of nano carbon powder. Carbon powder particles and the PVDF matrix form micro capacitors, which in turn establish a micro capacitor network. The presence of micro capacitors and their network significantly elevate the dielectric constant of the CP/PVDF composites. This interfacial polarization phenomenon, often referred to as the Maxwell–Wagner effect, manifests distinct behaviors [40,41]. As the content increases, the distribution of nano carbon powder particles becomes more extensive, leading to a further augmentation of micro capacitors and the micro capacitor network. Consequently, the dielectric constant exhibits a continuous increase with the rising content of the functional phase.

The CP/PVDF sample containing 10 wt% functional phase exhibits a remarkable permittivity of approximately 1589 at 800 kHz. This value represents a 450-fold increase compared to that of the pristine PVDF matrix (permittivity of 3.5). This significant increase in the permittivity can be attributed to the Maxwell–Wagner interfacial polarization effect. Due to their small dimensions and high specific surface area, the abundant nano carbon powder within the 10 wt% CP sample establish numerous interfacial capacitors with the surrounding PVDF matrix. These interfacial regions contribute significantly to the overall permittivity of the composites. However, this high permittivity comes at the expense of an elevated dielectric loss tangent of approximately 0.85. This loss likely originates from a combination of two mechanisms: the intrinsic polarization loss of the PVDF matrix and the conductive loss associated with the nano carbon powder [42]. It is noteworthy that the 5 wt% CP/PVDF composite displays a more favorable balance of permittivity and loss at 800 kHz. This sample exhibits a permittivity of 31.5, accompanied by a significantly lower dielectric loss tangent below 0.2. The reduced loss in this case can be ascribed to the lower content of the functional phase. With less nano carbon powder, the composite has a diminished interfacial area between the conductive phase and the PVDF matrix. This, in turn, leads to a decrease in conductive loss, allowing the intrinsic polarization loss of the PVDF substrate to become the dominant contributor to the overall dielectric loss.

## 3. Materials and Methods

### 3.1. Materials Preparation

Nano carbon powder (CP) with a purity greater than 99.5% and a diameter ranging from 30 to 40 nm was acquired from Aladdin Co. Ltd., Shanghai, China, with the product number C109965-5g. Polyvinylidene fluoride (PVDF) with a purity exceeding 99% was obtained from Shanghai Haoxi Nanotech, Co. Ltd., Shanghai, China. In this study, a hot-pressing technique was employed to fabricate CP/PVDF composites. Samples with seven different mass fractions of nano carbon powder were prepared, with the carbon-powder content increasing by 2.5% sequentially, labeled as 2.5, 5, 7.5, 10, 12.5, 15, and 17.5 wt%. The CP and PVDF powders were meticulously combined using a high-energy ball-milling apparatus to ensure homogenous dispersion of the CP within the PVDF matrix. The well-mixed powder blend was then transferred into a hot-pressing mold and subjected to a controlled heating and pressing cycle. The temperature was elevated to 160 °C to facilitate proper melting of the PVDF matrix, while a pressure of 15 MPa was applied for 15 min to promote densification of the composite and enhance interfacial bonding between the CP and PVDF phases. Following the hot-pressing cycle, the mold was cooled to room temperature at a controlled rate. The chosen mold has an inner diameter of 20 mm. Prior to hot pressing, an equal mass of mixed powder was weighed to ensure that the thickness of each sample remains essentially consistent. This process yielded disc-shaped samples with a diameter of approximately 20 mm and a thickness between 1 and 2 mm, thus meeting the test fixture requirements for a specimen diameter from 10 to 56 mm and a thickness of less than 10 mm.

### 3.2. Materials Characterization and Electrical Measurement

The Fourier-transform infrared (FTIR) measurements were conducted using a Nicolet 460 instrument from Thermo Fisher Scientific (Waltham, MA, USA). The Bruker D8 Advance diffractometer from Bruker Corporation (Billerica, MA, USA) was employed for X-ray diffraction (XRD) analysis to elucidate the phase composition of the composites. Raman spectroscopy was employed for further compositional analysis using a Renishaw RM2000 system which is from Renishaw plc (Gloucestershire, England, UK). Scanning electron microscopy (SEM) analysis was carried out on a Hitachi SU-70 instrument from Hitachi, Ltd. (Tokyo, Japan) to gain insights into the microstructure and morphology of the samples. The dielectric properties of the composites were evaluated using a high-precision Keysight E4980AL LCR meter equipped with a 16451B fixture (Santa Rosa, CA, USA). The fixture utilizes a parallel plate method to clamp the sample between two electrodes to form a capacitor. It is used in conjunction with the LCR meter to measure the capacitance; impedance; and other dielectric parameters, such as loss tangent. Measurements were conducted across a wide frequency range, spanning from 20 Hz to 1 MHz, employing a low-test voltage of 100 mV to minimize material distortions. This comprehensive approach enabled the direct acquisition of key electrical parameters, including capacitance (*C*p), resistance (*R*p), impedance (*Z*), and loss tangent (tanδ). The dielectric loss angle (δ), also known as the dielectric phase angle, is the angle between the phase of the electric field and the phase of the dielectric displacement in a dielectric material subjected to an alternating electric field. It quantifies the ability of a material to convert electric field energy into heat. A larger dielectric loss angle indicates greater energy loss and a more lossy material. The dielectric loss tangent (tanδ) is the tangent of the dielectric loss angle and is a dimensionless quantity that also measures the material’s loss. A higher dielectric loss tangent corresponds to a more lossy material. The dielectric loss tangent is calculated using the following formula:

(8) $$\tan\delta =\frac{\varepsilon_{r}^{\prime }}{\varepsilon_{r}^{\prime \prime }}$$

where $\varepsilon_{r}^{\prime }$ is the real part, and $\varepsilon_{r}^{\prime \prime }$ is the imaginary part of the relative dielectric constant.

Subsequently, the complex permittivity (the real part, $\varepsilon_{r}^{\prime }$; and imaginary part, $\varepsilon_{r}^{\prime \prime }$) and AC conductivity ($\sigma_{\mathrm{ac}}$) are calculated based on the obtained data. The calculation equations are as follows:

(9) $$\varepsilon_{r}^{\prime }=\frac{dC_{p}}{S\varepsilon_{0}}$$

(10) $$\varepsilon_{r}^{\prime \prime }=\frac{d}{2\pi {fsR}_{p}\varepsilon_{0}}$$

(11) $$\sigma_{\mathrm{ac}}=\frac{d}{R_{p}A}$$

The thickness of the sample is represented by the symbol *d*. *S* is the electrode area, *f* is the electric field frequency, and *A* is the electrode–sample contact area.

## 4. Conclusions

In this paper, CP/PVDF percolating composites were successfully prepared using the hot-pressing method, and a transition in the permittivity from positive to negative was observed. For carbon-powder contents of 12.5 wt% and higher, a transition to negative permittivity is observed in the composites. This behavior stems from the collective motion of free electrons within the established conductive network of the nano carbon powder, a phenomenon referred to as plasma oscillation. The AC conductivity analysis reveals that a percolation phenomenon occurred in the composites, which is also confirmed by microstructure characterization. The percolation threshold of the CP/PVDF composites is between 10 and 12.5 wt%. The negative permittivity in these composites can be effectively tailored by manipulating the carbon-powder content, highlighting the potential for controlling this property. Intriguingly, CP/PVDF composites with 5 wt% carbon powder achieve a remarkable balance between high permittivity and low dielectric loss. The development of CP/PVDF percolating composites with tunable negative permittivity represents a significant breakthrough. This paves the way for their use in diverse electromagnetic applications, including shielding, antennas, and beyond.

## Figures and Tables

**Table 1:** Comparison of negative-permittivity properties of the CP/PVDF composites with some polymer-based composites with nano carbon functional phase.

Composites	Filler Content	Test Frequency	Negative Permittivity	Ref.
Carbon nanotubes/epoxy	12 wt%	1–1000 MHz	~−100	[18]
Graphene/poly (vinyl alcohol)	10 wt%	10–1000 kHz	~−500	[15]
Graphene/polyaniline	2 wt%	100–1000 MHz	~−50	[34]
Graphene–carbon nanotubes/ PVDF	14 wt%	100–1000 MHz	~−25	[5]
Acetylene black/polyimide	24 wt%	10–1000 kHz	~−350	[38]
Nano carbon powder/PVDF	12.5 wt%	10–1000 kHz	~−4000	This work

## Data Availability

Data presented in this study are available upon request from the corresponding author.
